# Research progress and perspectives of noncoding RNAs in adrenocortical carcinoma: A review

**DOI:** 10.1097/MD.0000000000036908

**Published:** 2024-01-26

**Authors:** Changfen Xu, Peiyao Xu, Jiaqi Zhang, Sheng He, Tingting Hua, Aiwu Huang

**Affiliations:** a Department of Gynecology and Obstetrics, Hangzhou Lin’an TCM Hospital, Lin’an District, Hangzhou, China.

**Keywords:** adrenocortical carcinoma (ACC), biomarker, circular RNAs (circRNAs), long non-coding RNAs (lncRNAs), microRNAs (miRNAs)

## Abstract

Adrenocortical carcinoma (ACC) is a rare and highly aggressive endocrine malignancy. Although surgery can cure localized disease, but the majority of patients experience recurrence of ACC. The 5-year survival rate of patients with metastatic ACC is <15%, and the prognosis is poor. Therefore, it is urgent to explore the potential diagnostic markers and therapeutic targets for ACC. Recently, it has been proved that non-coding RNA (ncRNAs) is widely involved in pathological and physiological processes, including tumorigenesis and development. Aberrantly expressed ncRNAs have been found to be involved in the pathogenesis of ACC. Here, we summarized the expression patterns and the molecular mechanism of the involvement of microRNAs (miRNAs), long non-coding RNAs (lncRNAs) and circular RNAs (circRNAs) in ACC development. To explore the clinical value of ncRNAs as noninvasive biomarkers of ACC, we also displayed the relationship between the expression level of ncRNAs and the diagnosis and prognosis of patients with ACC.

## 1. Introduction

Adrenocortical carcinoma (ACC) is a rare malignancy characterized by invasive biology and underlying endocrine activity.^[1–3]^ The incidence of ACC is approximately 0.7 to 2.0 per million people, with a bimodal age distribution and a slightly higher incidence in women than that in men.^[4,5]^ Most ACC patients have no obvious early symptoms and invaded surrounding tissues or organs at the time of diagnosis, and even have distant metastases.^[6,7]^ In the majority of ACC patients, overproduction of estrogen, androgens, glucocorticoids, or aldosterone are observed.^[8,9]^ Some ACC patients present with fever or local symptoms such as fullness, pain, or a significant mass, while nonfunctional tumors may not be detected until the disease has advanced.^[10,11]^ Surgical resection is the main treatment for ACC patients with no infiltration or metastasis.^[12,13]^ For patients who have developed peripheral tissue infiltration, distant metastasis, or tumor recurrence, surgical resection combined with adjuvant therapy are the primary treatment, although, the effects are often more adverse.^[14–16]^ The overall prognosis of ACC remains poor, and the 5-year survival rates are about 30%.^[13]^ Only a few patients with ACC are associated with familial genetic disorders, while disseminated cases are associated with mutations in various genes such as tumor protein p53, catenin beta like 1, menin 1, and cyclin dependent kinase inhibitor 2A.^[17–19]^ However, the specific pathogenic mechanism of ACC remains unclear. Hence, exploring the diagnostic markers and therapeutic targets for ACC is urgent.

Genomic DNA is transcribed to produce 2 main types of RNAs, including coding RNAs and non-coding RNAs (ncRNAs).^[20–22]^ NcRNAs are derived from the genome and can be classified into 3 types according to their length.^[23,24]^ Those longer than 200 nucleotides are called long non-coding RNAs (lncRNAs),^[25]^ those with <200 nucleotides length are called medium-stranded ncRNAs,^[26]^ and those with approximately 20 nucleotides are called small non-coding RNAs. The importance of ncRNAs in the human genome cannot be overstated, as they comprise a significant majority of its composition. Advancements in high-throughput sequencing technologies have facilitated the identification of an increasing number of ncRNAs in eukaryotic cells.^[27]^ In accordance with well-established classification criteria, ncRNAs exhibit a diverse range of categories. Firstly, ncRNAs are divided into housekeeping ncRNAs, such as rRNAs and tRNAs, which carry out fundamental cellular functions, and regulatory ncRNAs, including miRNAs, circRNAs, and lncRNAs, which contribute to gene expression regulation.^[28]^ Secondly, the size of ncRNAs determines their classification, distinguishing between lncRNAs that exceed 200 nucleotides in length, and small ncRNAs that are below 200 nucleotides. Notable examples of small ncRNAs include miRNAs, small interfering RNAs (siRNAs), and piwi-interacting RNAs (piRNAs).^[29]^ Further classifications of lncRNAs exist based on their structural characteristics, delineating them as either linear lncRNAs or circular lncRNAs.^[30]^ Another classification criterion is the role of lncRNAs in gene expression regulation, which subsets them as either cis-lncRNAs, acting on nearby genomic loci, or trans-lncRNAs, exerting regulatory effects on distant genetic regions. Moreover, ncRNAs exhibit variability in subcellular localization, with examples including small nuclear RNAs and cytoplasm-located siRNAs. Additionally, ncRNAs can be classified based on their genomic origins, encompassing sense or antisense ncRNAs, bidirectional ncRNAs, intronic ncRNAs, and intergenic ncRNAs.^[31–33]^ The systematic and scientific classification of ncRNAs holds considerable significance in facilitating a comprehensive understanding of their characteristics. Recent studies have identified a new class of circular functional ncRNA-circular RNAs (circRNAs).^[34]^ CircRNAs are a type of single-chain, covalently closed ncRNAs with a 5′ cap structure or 3′ terminal poly(A) tail.^[35]^ In the past years, ncRNAs were considered “coding junks.” Recently, researchers have identified that ncRNAs are participate in transcriptional and posttranscriptional regulation, chromosome duplication, genomic blotting, RNA processing, selective splicing and modification, messenger RNA translation and stability,^[21,26,36–39]^ protein translocation and degradation functions,^[40–42]^ and the basic biochemical activities of organisms.

In this review, we summarized the roles and molecular mechanisms of ncRNAs in the ACC and might provide new perspectives for early diagnosis and targeted intervention for ACC.

## 2. Overview of ncRNAs

### 2.1. Biogenesis and function of miRNAs

**Figure 1.**
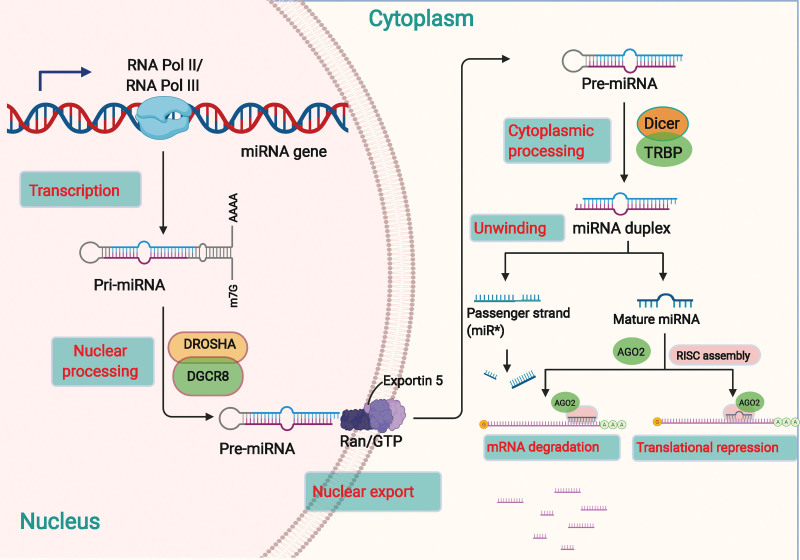
The biogenesis and biological function of miRNAs. The transcription of a pri-miRNA is regulated by RNA Pol II or III, while the nuclear processing of pri-miRNA become a pre-miRNA can be processed by DROSHA and DGCR8. Exportin 5 is involved in the processing of the nuclear export of the pre-miRNA. The cytoplasmic processing that pre-miRNA become a mature miRNA duplex is regulated by Dicer and TRBP. The miRNA duplex includes the passenger strand and the mature miRNA. The passenger strand is degraded, and the mature miRNA strand is integrated into the RISC to mediate either translational repression or mRNA degradation according to the extent of complementarity to the mRNA target. DGCR8 = DGCR8 microprocessor complex subunit; DROSHA = drosha ribonuclease III; miRNAs = microRNAs; Pol II = polymerase II; pre-miRNA = precursor miRNAs; pri-miRNA = primary miRNA; RISC = RNA-induced silencing complex.

MiRNAs are small non-coding RNAs with a length of about 22 bases.^[43]^ The biogenesis of miRNAs is a well-delineated conserved process, as displayed in Figure 1. Most miRNAs are first transcribed by RNA polymerase II (Pol II) in the nucleus to form the primary miRNA (pri-miRNA), a primary transcript with a hairpin-like structure.^[44–46]^ Pri-miRNA has a localized stem-loop structure with embedded mature miRNAs.^[46]^ A typical pri-miRNA is 33 to 35 bp long with a 5′ and 3′ region at the tail end and a single-stranded portion of the stem-loop, containing a 7-methylguanosine region at the 5′ end and a poly tail at the 3′ end.^[47]^ RNAse III-Drosha initiates miRNA maturation by shearing the stem-loop structure of pri-miRNA to release precursor miRNA (pre-miRNA), an RNA with a hairpin-like structure of approximately 70 bases in length.^[48]^ Exportin 5 binds to Ran-GTP to form a complex, wraps around the region of the pre-miRNA stem, and recognizes the 2 bases at the 3′ overhang of the pre-miRNA.^[49,50]^ This transport complex exits the nucleus and is hydrolyzed by GTP, leading to the disassembly and release of the pre-miRNA into the cytoplasm. The RNAse endonuclease III-Dicer then acts on the pre-miRNA to produce a miRNA duplex.^[51]^ The RNA-induced silencing complex (RISC) is formed by these duplexes bound to Argonaute proteins, and 1 strand is selected as the mature miRNA.^[52]^

MiRNAs can silence target mRNAs in the cytoplasm and inhibit the expression of target genes.^[53–55]^ They can also act in the nucleus by binding to the promoter region of target genes, ultimately silencing or overexpressing them.^[56,57]^ MiRNAs can even be encapsulated in exosomes and secreted extracellularly to biologically act on other cells.^[53,58]^ An increasing number of miRNAs are being discovered with the development of high-throughput sequencing technology. Ago2 and its family of proteins are thought to be the only RNA-binding proteins (RBPs) that interact with mature miRNAs.^[52]^ In addition, mature miRNAs also interact with proteins including heterogeneous nuclear ribonucleoprotein D, Hu antigen R (HuR), and others, allowing them to play a role in miRNA-mediated gene silencing.^[59]^ MiRNAs can modulate multiple genes, conversely, 1 gene can be regulated by multiple miRNAs, suggesting synergistic regulation between miRNAs and mRNAs. In addition, lncRNAs and circRNAs contain many miRNA-binding sites. LncRNAs or circRNAs can compete with downstream target genes though binding to miRNAs, which leads to a reduced suppressive influence of miRNAs on downstream target miRNAs and indirectly promotes the expression of downstream target genes.^[58]^

### 2.2. Biogenesis and function of lncRNAs

LncRNAs are a type of non-coding RNA molecules with transcripts of more than 200 nucleotides long.^[60,61]^ LncRNAs are linear RNA molecules that have two-dimensional (2D) and three-dimensional (3D) structures. Genes encoding lncRNAs are generally distributed within the genome, located in exons or introns of genes encoding mRNAs.^[62,63]^ LncRNA sequences are generally poorly conserved but their promoter regions and secondary structures are evolutionarily conserved.^[64,65]^ Most of the currently identified lncRNAs are produced by RNA Pol II transcription, and the vast majority of them are non-coding.^[66,67]^ Although lncRNAs are transcribed from various genomic locations, they can be categorized into 5 distinct classes based on their origin.^[68–70]^ These classes include sense, antisense, bidirectional, intronic, and intergenic lncRNAs. Sense lncRNAs are transcribed from the same strand as the associated protein-coding gene, while antisense lncRNAs are transcribed from the opposite strand. Bidirectional lncRNAs are located near the promoter region of the associated protein-coding gene and transcribe in the opposite direction. Intronic lncRNAs originate from long introns within the protein-coding gene and intergenic lncRNAs arise from the intergenic regions between 2 protein-coding genes. Different transcriptional activation mechanisms, poly(A) modifications, and expression patterns may be present among these different types of lncRNAs within the cell.

**Figure 2.**
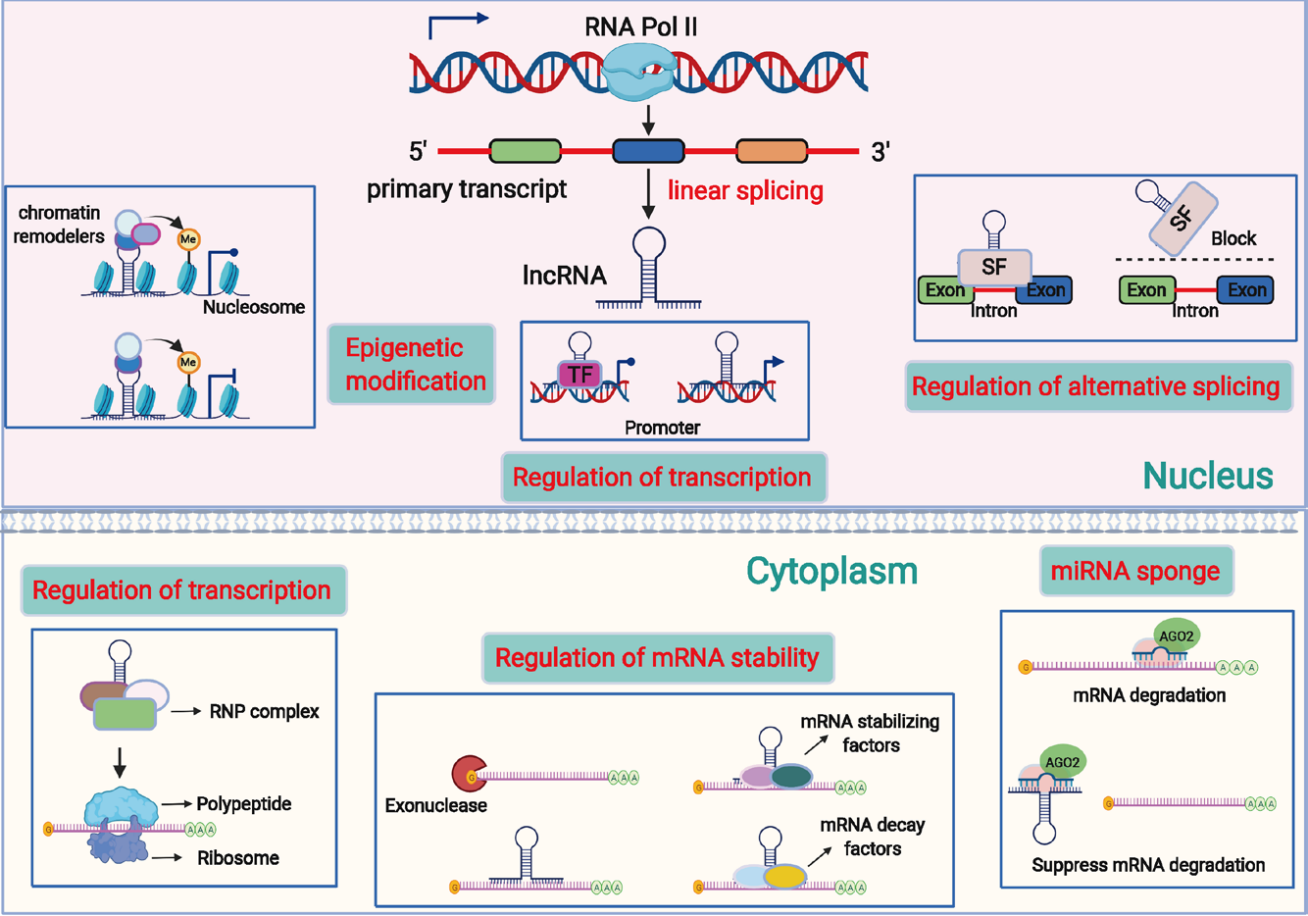
The regulation mechanism of lncRNAs. An overview of the role of lncRNA in the initiation and progression of cancers by regulating downstream gene expression. In the nucleus, lncRNAs can bind to chromatin remodelers and histone to regulate gene expression. LncRNAs can bind to different TFs to the gene promoter to promote or repress transcription. LncRNAs can also directly bind to the promoter of downstream gene and enhance its transcription. Besides, lncRNAs can interact with SFs and guide them to carry out the processing of alterative splicing of the pre-mRNAs, and so as to generate different mRNA variants. In the cytoplasm, lncRNAs can act as competitive endogenous RNAs (ceRNAs) to competitively bind to the miRNAs and contribute to the free of target mRNAs. LncRNAs can also participate in the regulation of mRNA stability. They can directly bind to the mRNAs to form RNA-RNA duplex to protect them from degradation by endo- or exonucleases. While some lncRNAs can bind with RBPs to promote or suppress the stability of target mRNAs. Moreover, lncRNAs can interact with RBPs, RNPs and translation factors to regulate translation of target mRNAs or directly encode peptide. ceRNAs = competitive endogenous RNAs; lnRNAs = long noncoding RNAs; pre-mRNAs = precursor mRNAs; RBPs = RNA binding proteins; RNPs = ribonucleoproteins; SFs = splicing factors; TFs = transcription factors.

LncRNAs play crucial regulatory roles in biological processes through a variety of molecular modalities, including interactions with DNA, RNA, proteins, and polypeptides^[71–74]^ (Fig. 2). They can bind to DNA to alter chromatin structure and take part in the regulation of epigenetic modifications, thereby influencing the expression of target genes.^[40,75]^ LncRNAs can also act as competitive endogenous RNAs by binding to both mRNAs and miRNAs to indirectly adjust the expression of downstream target genes. Briefly, lncRNAs have the following main functions, such as miRNA sponging, protein binding, DNA binding, transcriptional regulation, and encoding small peptides. LncRNA PVT1 may promote gemcitabine resistance in pancreatic cancer by modulating the autophagy and Wnt/β-catenin pathways by regulating the miR-619-5p/autophagy related 14 and miR-619-5p/pygopus family PHD finger 2 signaling pathways.^[76]^ LncRNAs can bind to several RBPs and take part in a variety of biological processes by affecting protein stability, subcellular localization, and protein complex formation. The oncogenesis and progression of tumors can be inhibited by enhancing the binding of beta-transducin repeat containing E3 ubiquitin protein ligase, a ubiquitin E3 ligase, to HuR, thereby lowering the levels of HuR and its target mRNA.^[77]^ Nuclear lncRNAs can bind to non-coding or protein-coding regions through complementary base pairing and regulate target gene expression in a cis or trans manner. Lnc-HOXA11-AS can interplay with WD repeat domain 5 and accelerate β-catenin transcription, bind with enhancer of zeste 2 polycomb repressive complex 2 subunit, suppress p21 transcription, and induce Kruppel like factor 2 mRNA degradation by interplaying with Staufen double-stranded RNA binding protein 1.^[78]^ Previously, researchers believed that lncRNAs do not have coding potential, which has since been disproved. For example, lncRNA HOXB-AS3 encodes a conserved peptide HOXB-AS3-53 aa, which can antagonize the RGG motif of heterogeneous nuclear ribonucleoprotein A1 by competing for binding to the arginine residue in the RGG motif. Exon 2 of lncRNA P53 induced transcript can be looped and translated into an 87 amino acid peptide, PINT87aa,^[79]^ which binds to the DNA binding domain of forkhead box protein M1 and participates in mitochondrial autophagy.^[80]^

### 2.3. Biogenesis and function of circRNAs

**Figure 3.**
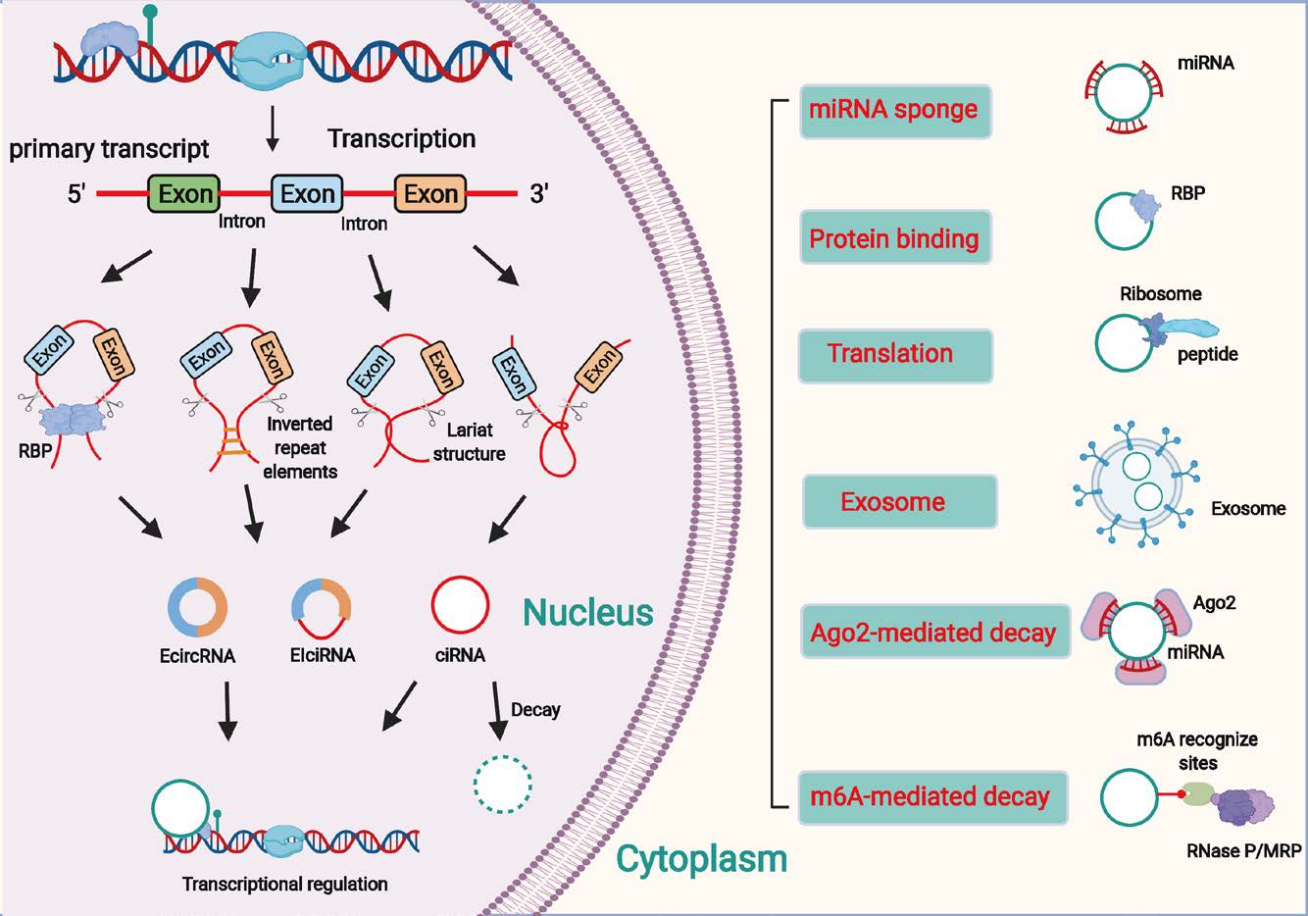
The biology of circRNAs. Most circRNAs are generated from pre-mRNAs by various back-splicing processes. CircRNA maturation go through which competes with the traditional mRNA splicing. In basis of the mechanism of synthesis, circRNAs can be classified into EcircRNAs, ciRNAs and EIciRNAs. In the nucleus, circRNAs can participate in the regulation of transcription. In the cytoplasm, circRNAs are involved in many biological processes such as miRNA sponging, RBP binding and translation. CircRNAs can be degraded via Ago2-mediated decay and m6A-mediated decay. Besides, circRNAs are enriched and stable in exosomes and play an essential role in various biological processes. Ago2 = argonaute RISC catalytic component 2; circRNAs = circular RNAs; ciRNAs, intron circRNAs; EcircRNAs = exon circRNAs; EIciRNAs, exon-intron circRNAs; pre-mRNAs = precursor mRNAs; RBPs = RNA binding proteins.

The biogenesis and biological function of circRNAs is showed in Figure 3.

CircRNAs are a novel class of ncRNAs formed by the ligation of mRNA 3′ and 5′ ends, primarily generated from introns or exons by reverse splicing or lassoing introns.^[81–83]^ Advanced sequencing technologies have revealed that circRNAs are widely and steadily expressed in eukaryotic cells and play an important role in the regulation of gene expression in human cells.^[84–86]^ CircRNAs are mainly formed by intron and exon cyclization.^[87]^ Based on the mechanism of synthesis, circRNAs can be classified into exon, intron, and exon–intron circRNAs.^[88,89]^ On the mRNA precursors, successive assemblies of small ribosomal proteins catalyze the attachment of the 5′ donor site downstream of the exon to the 3′ acceptor site upstream, and the first and last insertions cyclize by shearing to form circRNAs.^[90,91]^ Some circRNAs contain reverse complementary sequences in introns on both sides of the exon, which form RNA duplexes at the shear site and form 2 types of circRNAs, those with and without introns by variable shearing.^[92]^ In addition, the formation of circRNAs can be facilitated by binding RBPs to introns flanking exons.^[93,94]^ Unlike lncRNAs, which can have linear structures with a 5′ cap and 3′ poly(A) tail, circRNAs are covalently closed single-stranded RNA molecules lacking these structures. While circRNAs are mainly generated through reverse splicing or lassoing of introns or exons, lncRNAs have a more diverse origin and can be transcribed from various regions of the genome.^[95]^ This structural feature makes circRNAs highly stable and resistant to digestion by ribonucleases. In addition, circRNAs mostly comprise exons, although a few are formed by intron cyclization, which is highly conserved in interspecies evolution; such circRNAs have temporal specificity in certain tissue cell sources and different developmental stages.^[96,97]^

Both lncRNAs and circRNAs exert their biological roles by binding to DNA, RNA, or proteins. They can act as intracellular competitive endogenous RNAs to regulate gene expression by directly interacting with miRNAs. They can also modulate target gene expression by altering the binding of transcription factors to promoters. In addition, they can facilitate target genes’ activation or silencing by forming scaffolding complexes with effector molecules.^[98,99,100,101]^ Circular RNA erbb2 interacting protein (circ-ERBIN) regulates the expression of hypoxia-inducible factor (HIF)-1α by binding to miR-125a-5p and miR-138-5p. Circ-ERBIN can accelerate cap-independent protein translation of HIF-1α, and promote colorectal cancer proliferation, migration, and metastasis in vitro and in vivo.^[102]^ CircRNAs are also involved in the regulation of gene transcription through multiple pathways. Some exon-intron circRNAs can interact with U1 small ribonucleoprotein and promote parental gene transcription by binding to RNA Pol II.^[100,103]^ The splicing factor exons can be cyclized to form circRNAs, which compete with the linear splicing of precursor mRNAs to influence the formation of linear RNAs and play a role in regulating the expression of related genes.^[81,104]^ Recent studies have also shown that circRNAs can also encode proteins with translational functions.^[103]^ For instance, circPPP1R12A can encode a functional protein and has an open reading frame, circPPP1R12A-73aa, which promotes cervical cancer (CC) growth and metastasis by activating the Hippo-YAP signaling pathway.^[105]^

## 3. ncRNAS and ACC

### 3.1. MiRNAs and ACC

Since their discovery, several miRNAs have been identified in a variety of tissues and organisms. MiRNAs are now known as pivotal mediators that can affect almost all biological processes, including development, immune response, cell proliferation and differentiation, tumor initiation, and metastasis.^[106,107]^ Many miRNAs are aberrantly expressed in ACC tissues and are closely related to the onset and progression of ACC (Table 1).

**Table 1:** Functional characterization of noncoding RNAs in ACC.

Noncoding RNA	Expression	Role	Function role	miRNAs	Related genes	Reference
MiRNAs						
miR-7-5p	Downregulated	Tumor suppressor	Reduce cell proliferation and induce cell cycle arrest	/	RAF1 and CDK1	Glover et al
miR-99a and miR-100	Upregulated	Oncogene	Promote cell growth and proliferation	/	IGF-mTOR signaling	Doghman et al
miR-195	Upregulated	Oncogene	Promote cell proliferation, migration, invasion and adhesion	/	ZNF367/ITGA3	Jain et al
miR-205	Downregulated	Tumor suppressor	Impair cell proliferation and induce cell apoptosis	/	Bcl-2	Wu et al
miR-431	Downregulated	Tumor suppressor	Decrease the concentrations of doxorubicin and mitotane and increase apoptosis	/	ZEB1	Kwok et al
miR-483-5p	Upregulated	Oncogene	Promote cell invasion and progression	/	NDRG2	Agosta et al
miR-139-5p					NDRG4	
miR-486-5p	Downregulated	Tumor suppressor	Inhibit cell proliferation		FASN	Li et al
LncNAs						
ASB16-AS1	Downregulated	Tumor suppressor	Inhibit cell proliferation	/	HuR/IGF1R/CDK6	Buishand et al
HOTAIR	Upregulated	Oncogene	Promote cell proliferation and cell cycle	/	Cyclin D1/p-Rb/p-GSK3β	Glover et al
LINC00271	Downregulated	Tumor suppressor	Arrest cell cycle	/	Wnt signaling	Hassan et al
MALAT1	Upregulated	Oncogene	Promote cell proliferation and cell cycle	miR-497	EIF4E and SFPQ	Guo et al
UCA1	Upregulated	Oncogene	Promote cell proliferation and inhibit cell apoptosis	miR-298	CDK6	Yan et al
CircRNAs						
circ-CCAC1	Upregulated	Oncogene	Promote cell proliferation, migration and invasion	miR-514a-5p	C22orf46	Ansari et al

/ = no data, ACC = adrenocortical carcinoma, miRNAs = microRNAs.

#### 3.1.1. The biological roles of miRNAs in ACC.

The prognosis of ACC is poor, and the overall survival (OS) rate of ACC remains unsatisfied due to the late diagnosis, high recurrence/metastasis rate, and poor response to conventional therapy. As miRNAs are being studied more intensively, new ideas for the treatment of ACC have emerged. MiR-7-5p expression was significantly reduced in ACC and could suppress cell proliferation and contribute to cell cycle arrest by targeting rapidly accelerated fibrosarcoma 1 and mammalian target of rapamycin.^[108]^ Targeted delivery of extracellular vesicles containing miR-7-5p in vitro significantly inhibited ACC growth, indicating the potential of miR-7-5p to be a novel therapy for ACC. MiR-205 was greatly less expressed in ACC tissues than in adrenocortical adenoma (ACA) and that miR-205 could inhibit apoptosis by targeting the apoptotic gene Bcl-2 to activate Bcl-2/Bax/caspase3/9.^[109]^ MiR-205 may be implicated in the regulation of ACC growth as an oncogene. Kwok and colleagues tested the expression of miRNAs in samples of primary ACC tumors from 10 stage IV patients and found under-expressed miR-431 in patients with progressive disease ACC receiving adjuvant therapy. Overexpression of miR-431 in cells could significantly reduce the half-maximal inhibitory concentrations of adriamycin and mitotane and significantly promoted apoptosis. MiR-431 could regulate the cellular epithelial-mesenchymal transition by targeting Zinc Finger E-Box Binding Homeobox 1.^[110]^ This suggested that synthesis of miR-431 and delivery it to circulating blood might significantly improve therapeutic efficacy in advanced ACC. To identify early molecular alterations, li and colleagues collected adrenal glands from young diversiform endocrine tumor type 1 conventional knockout mice (Men1+/−) that closely mimic human multiple endocrine neoplasia type 1 features. The results of detection and analysis revealed that under-expressed miR-486-3p could significantly inhibit tumor growth. Mechanistic studies revealed that suppression of miR-486-3p might promote proliferation by increasing fatty acid synthase, thus promoting fatty acid production.^[111]^

MiRNAs can also act as pro-oncogenes to promote the malignant progression of ACC. MiR-99a and miR-100 were highly expressed in ACC and they could contribute to malignant growth and progression of ACC by regulating IGF-mechanistic target of rapamycin kinase (mTOR) signaling, thus promoting the cell cycle.^[112]^ Pharmacological inhibition of mTOR signaling using everolimus greatly decreased tumor cell growth in vivo and in vitro. Zinc finger protein 367 (ZNF367) was observed to be overexpressed in ACC, and its overexpression in ACC cell lines observably inhibited cell proliferation, invasion, migration, and adhesion of extracellular proteins.^[113]^ Bioinformatic analysis verified that miR-195 could directly elevate ZNF367 expression by binding to the 3′ UTR of ZNF367, which in turn regulated integrin subunit alpha 3 expression. Thus, miR-195/ZNF367/ITGA3 might be a latent therapeutic target for ACC. Moreover, miR-483-5p/NDRG family member 2 and miR-139-5p/NDRG4 pathways could accelerate ACC invasiveness by mediating the epithelial–mesenchymal transition process, with potential and prognosis intervention in adrenocortical malignancies.^[114]^

#### 3.1.2. Diagnostic value of miRNAs in ACC.

The etiology of ACC is not fully understood, and traditional screening methods include magnetic resonance imaging, computerized tomography (CT), and 18 positron emission tomography (18F PET-CT).^[13,115]^ MiRNAs have emerged to provide new ideas for the diagnosis of ACC (Table 2). The relative expression of miRNAs in serum samples from 17 patients with ACC and 22 patients with ACA were measured by quantitative real-time polymerase chain reaction (qRT-PCR). MiR-34a and miR-483-5p were significantly higher in the serum of patients with ACC than in those with ACA. MiR-34a and miR-483-5p had latent diagnostic value in the early stages of ACC. The area under the ROC curve (AUC) for miR-34a as a diagnostic marker was 0.8102, and the AUC for miR-483-5p was 0.7406.^[116]^ Another study evaluated the expression levels of 4 miRNAs in tissue specimens from 26 patients with ACA and 10 patients with ACC and showed that miR-100, miR-125b, and miR-195 were observably downregulated, and miR-483-5p was upregulated in comparison to those in innocent tumors. Furthermore, miR-483-5p showed a high level of expression in differentiating benign from malignant adrenocortical tumors. To ascertain whether circulating miR-483-5p levels can distinguish patients with ACC with a good prognosis (no recidivation for at least 3 years) from those with a poor prognosis (recidivation or demise within 3 years after surgery), a single-center retrospective analysis was performed using sera from 48 patients with primitively nonmetastatic and surgically treated ACC. The analysis showed that a sensitivity of 61.5% and specificity of 100% for circulating miR-483-5p with a AUC of 0.853. In addition, and miR-483-5p offered the simplex best prognostic value for recurrence-free survival (risk ratio for recurrence (HR) of 5.98, *P* < .011) in a multivariate analysis.^[117]^ This suggested that serum miR-483-5p is a potent prognostic biomarker for early postoperative ACC. Decmann and colleagues also demonstrated that significant overexpression of miR-483-5p was observed in plasma samples from patients with ACC in comparison to patients with ACA (*P* < .0001, sensitivity: 87%, and specificity: 78.3%). Nevertheless, miR-483-5p was not detected observably in the urine samples of patients with ACC and ACA.^[118]^ MiRNA expression profiles in 51 samples (15 ACC samples, 18 ACA samples, and 18 control samples) was analyzed by next-generation sequencing. The results revealed that the expression levels of 6 miRNAs (miR-210, miR-421, miR-450a-5p, miR-483-3p, miR-483-5p, and miR-503-5p) predicted the sample status (malignant/nonmalignant) with at least 95% accuracy, with miR-483-5p having the highest diagnostic rate.^[119]^ Moreover, Chabre et al, measured the expression levels of 5 miRNAs in serum specimens from 56 subjects (19 healthy controls [HC], 14 ACA patients, 9 patients with nonaggressive ACC [naACC], and 14 patients with aggressive ACC [aACC]). The levels of miR-335 and miR-195 were significantly lower in tumor and serum samples from patients with ACC than in those with HC or ACA. The levels of miR-376a and miR-139-5p were significantly increased in patients with aACC compared to naACC in tumor samples merely. Tissue miR-483-5p was significantly upregulated in most ACC in comparison to HC patients or ACA, but most importantly, serum miR-483-5p was only detected in aACC patients. Low circulating levels of miR-195 or High circulating levels of miR-483-5p are related to shorter relapse-free survival and shorter OS.^[120]^ All above results demonstrated that miRNAs might be the potential diagnostic biomarkers in ACC.

**Table 2:** The potential of ncRNAs in the diagnosis and prognosis of ACC.

Study	Sample size	ncRNAs	Expression	Source	Expression (*P* value)	Diagnostic value	Sample size	Prognostic value		Reference
						AUC		RFS (*P* value)	OS (*P* value)	
Patel et al	(Benign: ACC)	miR-34a	Up	Serum	*P* = .0011	0.8102		/	/	Patel et al,.
	(22: 17)	miR-483-5p	Up	Serum	*P* = .0113	0.7406		/	/	
Patterson et al	(Benign: ACC) (26: 10)	miR-100	Down	Tissues	*P* < .05	0.717		/	/	Oreglia et al,.
		miR-125b	Down	Tissues	*P* < .05	0.763		/	/	
		miR-195	Down	Tissues	*P* < .05	0.771		/	/	
		miR-483-5p	Up	Tissues	*P* < .05	0.943		/	/	
Oreglia et al	(NR3yrs: *R* < 3yrs) (13: 13)	miR-483-5p	Up	Serum	*P* = .0025	0.853	ACC(n = 26)	*P* = .0005	*P* = .007	Decmann et al
Decmann et al	(ACA: ACC) (23: 23)	miR-483-5p	Up	Serum	*P* < .0001	0.876		/	/	Koperski et al
Koperski et al	(ACC: Benign) (8: 20)	miR-503-5p	Up	Tissues	*P* < .0001	1		/	/	Chabre et al
		miR-483-3p	Up	Tissues	*P* < .0001	0.987		/	/	
		miR-450a-5p	Up	Tissues	*P* < .0001	0.974		/	/	
		miR-542-5p	Up	Tissues	*P* < .0001	0.914		/	/	
		miR-542-3p	Up	Tissues	*P* < .0001	0.868		/	/	
		miR-424-5p	Up	Tissues	*P* < .0001	0.829		/	/	
		miR-210	Up	Tissues	*P* < .0001	1		/	/	
		miR-450b-5p	Up	Tissues	*P* < .0001	0.941		/	/	
		miR-483-5p	Up	Tissues	*P* < .0001	1		/	/	
		miR-421	Up	Tissues	*P* < .0001	0.954		/	/	
		miR-184	Up	Tissues	*P* < .0001	0.98		/	/	
		miR-424-3p	Up	Tissues	*P* < .0001	1		/	/	
		miR-128	Up	Tissues	*P* < .0001	0.961		/	/	
		miR-598	Up	Tissues	*P* < .0001	0.842		/	/	
		miR-148b-3p	Up	Tissues	*P* < .0001	0.954		/	/	
Chabre et al	(ACA: ACC) (10: 18)	miR-195	Down	Tissues	*P* < .05	0.83		/	/	Wang et al
		miR-335	Down	Tissues	*P* < .05	0.877		/	/	
		miR-483-5p	Up	Tissues	*P* < .01	0.904		/	/	
	(aACC: naACC) (9: 9)	miR-139-5p	Up	Tissues	*P* < .001	0.941		/	/	
		miR-376a	Up	Tissues	*P* < .05	0.899		/	/	
	(ACA: ACC) (14: 23)	miR-195	Down	Serum	*P* < .001	0.948	ACC(n = 21)	*P* = .0014	*P* = .0086	
		miR-335	Down	Serum	*P* < .01	0.837				
		miR-139-5p	Up	Serum	*P* < .05	0.714				
		miR-376a	Down	Serum	*P* < .05	0.811				
	(aACC: naACC) (14: 9)	miR-483-5p	Up	Serum	*P* < .01	0.929		*P* = .0004	*P* = .0005	
Long et al	(Benign: ACC) (19: 21)	lnc-ASB16-AS1	Down	Tissues	*P* = .003	/	ACC(n = 57)	/	*P* < .01	Buishand et al
Yan et al	(Benign: ACC) (30: 77)	lnc-HOTAIR	Up	Tissues	*P* < .001	/	ACC(n = 77)	/	*P* = .0028	Glover et al
Glover et al	(NRACC: RACC) (9: 11)	lnc-PRINS	Down	Tissues	*P* < .01	0.889	/	/	/	Xu et al
	(NMACC: MACC) (14: 5)	lnc-PRINS	Down	Tissues	*P* < .05	0.843	/	/	/	
Li et al	(Benign: ACC) (48: 48)	circ-CCAC1	Up	Tissues	*P* < .01	/	ACC(n = 48)	*P* = .006	/	Ansari et al

/ = no data, ACC = adrenocortical carcinoma, miRNAs = microRNAs.

### 3.2. LncRNAs and ACC

Dysregulation of lncRNA expression may result in abnormal cell function and tumor growth, which may be the main causes of diseases. LncRNAs also play a key role in cancers, and their abnormal expression, mutations, or single nucleotide polymorphisms are associated with tumorigenesis and metastasis.^[121–123]^ Here, we found that aberrant expression of lncRNAs is also an essential cause of tumorigenesis and malignant progression in ACC (Table 1).

#### 3.2.1. The biological roles of lncRNAs in ACC.

LncRNAs are involved in ACC progression by regulating several signaling pathways. Lnc-ASB16-AS1 was downregulated in ACC, and it could act as a tumor growth suppressor in vivo and in vitro. Transcriptome RNA sequencing identified insulin like growth factor 1 receptor and cyclin dependent kinase 6 (CDK6) in ACC cells as potential downstream targets of ASB16-AS1. Lnc-ASB16-AS1 promoted the binding of ubiquitin E3 ligase beta-transducin repeat containing E3 ubiquitin protein ligase to HuR, thus promoting the deubiquitination and degradation of HuR protein, thereby regulating the expression of IGF1R and CDK6 mRNA.^[124]^ HOX transcript antisense RNA (HOTAIR) was the first lncRNA identified with trans-transcriptional regulation. HOTAIR is closely related to tumors and may be involved in regulating tumor development. HOTAIR expression was discovered to be higher in ACC tissues than in normal tissues, and transfection of H295R cells with HOTAIR siRNA significantly suppressed cell proliferation and induced cell cycle arrest. Besides, LINC00271 expression was positively associated with cell cycle, Wnt signaling, and chromosome segregation pathways in ACC.^[125]^

Endogenous RNA molecules containing the same miRNA response elements, mainly lncRNAs, circRNAs, mRNAs, and pseudogene transcripts, can compete to bind to specific sites of the corresponding miRNAs to regulate cell growth, proliferation, and differentiation at the posttranscriptional level. In recent years, research on ACC based on ceRNA theory has significantly advanced and has deepened the understanding of ACC development from RNA-RNA interactions to the regulation of downstream biological effects. Metastasis associated lung adenocarcinoma transcript 1 (MALAT1) is one of the first identified lncRNAs, which is aberrantly expressed in a variety of tumors and closely related to tumorigenesis and progression. MALAT1 also has potential applications in the fields of early diagnosis, treatment, and prognosis assessment of tumors. MALAT1 expression was upregulated in ACC tissues, and it could compete with miR-497 to promote eukaryotic translation initiation factor 4E expression and thus to promote the cell cycle, and the MALAT1/miR-497/EIF4E regulatory axis might be a latent therapeutic target for ACC.^[126]^ Urothelial carcinoma antigen l is an important lncRNA that exhibits proto-oncogene function and is highly expressed in a variety of tumors, especially in bladder cancer. Guo and team members found that UCA1 was highly expressed in ACC tissues and was closely related to TNM staging and metastasis in patients with ACC. UCA1 overexpression observably accelerated ACC cell proliferation and inhibited apoptosis. Mechanistic studies suggested that UCA1 might act as a sponge for miR-298 to regulate CDK6 expression, which in turn to contribute to the malignant progression of ACC.^[127]^

#### 3.2.2. Diagnostic value of lncRNAs in ACC.

With the rapid progress of lncRNA research, especially the relationship between lncRNAs and ACC, an increasing number of lncRNAs have been regarded as the diagnostic tool fo ACC (Table 2). Long and colleagues detected the expression level of ASB16-AS1 in 57 specimens of ACC and found that the OS was shorter in patients with lower ASB16-AS1 expression than in those with higher ASB16-AS1 expression. In addition, the expression level of ASB16-AS1 correlated negatively with Ki-67 index, tumor size, tumor stage, lymph node metastasis, distant metastasis, and lymph node metastasis.^[124]^ The mRNA expression of HOTAIR in 77 ACC tissues and 30 normal adrenal tissues was detected by qRT-PCR. The results indicated that the expression level of HOTAIR was closely associated with the depth of tumor infiltration as well as TNM. In addition, disease-free survival was significantly lower in the high HOTAIR expression group.^[128]^ Glover and colleagues analyzed the lncRNA expression profile in the ACA, ACC, and normal adrenal cortex. A total of 956 lncRNAs were distinctively expressed between normal adrenal cortex and ACC, and 85 lncRNAs were distinctively expressed between ACA and ACC. Overall, they found that low expression of PRINS was closely related to ACC recurrence and metastasis and is a good diagnostic marker for ACC.^[129]^

### 3.3. CircRNAs and ACC

Many circRNAs are abnormally expressed in tumors and regulate tumor development, which, together with the stability and tissue specificity of circRNAs, suggest that circRNAs may become a new biomarker for diagnosis and a new therapeutic target in the future.

#### 3.3.1. The biological role of circRNAs in ACC.

Circ-CCAC1 is located on chr17: 37880978–37882106 and is looped by exons 23 to 26 of ERBB2. CCAC1 has previously been shown to be aberrantly expressed in CCA and is closely associated with tumorigenesis and progression.^[130]^ Li et al found that circ-CCAC1 was overexpressed in ACC tissue samples and cell lines and associated with poor prognosis. In vitro results showed that circ-CCAC1 could act as an oncogene in ACC, and circ-CCAC1 enhanced C22orf46 expression in ACC cells by up taking of miR-514a-5p^[131]^ (Table 1). To date, no studies on other circRNAs in the ACC have been reported.

#### 3.3.2. Diagnostic value of circRNAs in ACC.

In the cytoplasm of eukaryotic cells, circRNAs are abundant and have tissue-specific, time-of-action-specific, and disease-specific properties. Since most exosomal circRNAs are less than 1000 nt in length and mostly above 200 bases, they can be quantified by reverse PCR and are highly actionable. Therefore, circRNAs have the potential to be good diagnostic markers for diseases. The enrolled patients with ACC were divided into 2 groups (low and high expression groups) on the basis of the median value of circ-CCAC1. High circ-CCAC1 expression was found to be associated with poorer OS in patients with postsurgical ACC^[131]^ (Table 2).

## 4. Discussion and perspective

In recent years, with an increasing number of studies on ncRNAs, researchers have discovered that their mechanism of action are diverse, and their biological function are very powerful. NcRNAs exert vital roles in distinct physiological and pathological processes, making it a novel research hotspot in the field of epigenetics. MiRNAs are evolutionarily conserved and diverse and can only be expressed in specific tissues and developmental stages. Besides, it also plays significant regulatory roles in cell growth, development, and other physiological processes.^[132]^ MiRNAs can not only regulate intracellular gene expression but also act as antiviral factors against viral mRNAs in plants and invertebrates.^[133,134]^ In vertebrates, miRNAs not only resist viral mRNAs but also act as products of interference systems.^[135,136]^ LncRNAs can affect the activation and repression of gene expression through cis- and trans-actions and processes, such as chromatin modification and chromatin remodeling.^[137]^ Moreover, individual lncRNAs can exert regulatory functions through modular structural domains, often linking protein activity to DNA or RNA targets through interactions with them.^[73]^ To date, many diseases have reported to be associated with disorders of lncRNAs expression. With extensive research, the biological effects of lncRNAs and their molecular mechanisms will be further elucidated and provide new ideas and targets for the diagnosis and treatment of diseases. CircRNAs are the key roles involved in tumor initiation and progression, while many issues remain to be solved. For examples, exon circRNAs are constituted by reverse splicing; how the spliceosomes specifically recognize the exons of circRNAs, but not the exons of linear RNAs need to be examined. In addition, m6A-modified circRNAs usually originate from exons of unmethylated mRNAs, and circRNAs of exons of methylated mRNAs are less stable. Thus, it is not clear whether m6A modification affects the stability of circRNAs, how circRNAs are degraded, and what different functions the loop structure may confer on the linear RNAs corresponding to them.

Diagnostic and therapeutic applications of ncRNAs are rapidly evolving, and biomarkers and drugs based on ncRNAs may have clinical applications in the future. Norsworthy found that circulating miRNAs show high accuracy in differentiating sporadic Creutzfeldt-Jakob disease from Alzheimer disease.^[138]^ Tan and colleagues proved that HOTAIR levels in serum samples of glioblastoma (GBM) patients were significantly higher than corresponding controls, and the AUC for differentiating GBM patients from controls was 0.913, with a sensitivity of 86.1% and specificity of 87.5% at a cutoff value of 10.8.^[139]^ Hsa_circ_0001946 and hsa_circ_0043603 were reported to be good early diagnostic markers for esophageal squamous cell carcinoma (ESCC) by next-generation sequencing (NGS). In addition, hsa_circ_0001946 can also predict tumor recurrence, OS, and DFS of ESCC patients.^[140]^ Exosomes, as effective carriers of intercellular genetic material and other information communication, have become powerful tools for studying tumor pathogenesis and developing liquid biopsy techniques, which may be the new field for tumor diagnosis and treatment. MiR-106b-5p, miR-15a-5p, and miR107 were observably upregulated in plasma exosomes of patients with endometrial cancer (EC) compared to healthy subjects by high-throughput sequencing. MiR-15a-5p alone produced an AUC value of 0.813, and integration with CEA and CA125 resulted in an AUC value of 0.899. Exosomal-miR-15a-5p was also related to the depth of muscle infiltration and invasiveness of EC, as well as with reproductive hormone levels such as TTE and dehydroepiandrosterone.^[141]^ In a multicenter study, Guo et al found that circulating exosomal lncRNA-gastric cancer 1 could be used as a noninvasive biomarker to detect early gastric cancer and monitor disease progression, and combining circulating exosomal lncRNA-GC1 assay with endoscopy could improve the early diagnosis of GC.^[142]^ Xu and colleagues demonstrated that bile-resident exosome circ-CCAC1 functioned better as a diagnostic biomarker than serum-derived EVs from patients with cholangiocarcinoma.^[130]^ In our review, we summarized the applications and values of ncRNAs in the diagnosis of ACC. However, no studies on exosome-derived ncRNAs in ACC have been reported so far, and further researches are needed to explore them.

Furthermore, various of researches have indicated that ncRNAs play vital role in tumor therapy. The roles of ncRNAs in the mechanism of tumor drug resistance are crucial and have profound implications for the treatment of malignant tumors. Xin et al found that chemical modification of the 3′ end of miR-519c using 2′-methyl phosphorothioate (OME-PS) strengthened its activity and stability without inducing immunogenicity. The multifunctional nanodrug OME-PS-miR-519c significantly inhibited gemcitabine resistance in pancreatic cancer.^[143]^ LncSNHG12 could act as the sponge of miR-129 and improve the clinical efficacy of Temozolomide (TMZ) chemotherapy in GBM.^[144]^ Circ-SORE binds miR-660-3p and miR-103a-2-5p by acting as miRNA sponges. This competitively induces sorafenib resistance and activates the Wnt/β-catenin pathway, leading to the malignant progression of hepatocellular cancer.^[145]^ However, no studies on the roles of ncRNAs in the treatment of ACC have ever been reported. With the development of bioinformatics tools, an increasing number of novel ncRNAs relevant to the therapeutic and clinical applications of ACC remain to be discovered.

Currently, the detection of ncRNAs in tumors is mainly focused on tissue samples, which is a more invasive method and is not suitable for early clinical tumor diagnosis. Compared with tissues, clinical samples such as serum, urine, and body fluids are more easily accessible and noninvasive. In the future, methods to assess the expression of ncRNAs in serum and disease-related body fluids (gastric fluid, cerebrospinal fluid, plasma fluid, etc) should be further developed. Although a mass of studies have confirmed the pathogenic mechanisms of ncRNAs in ACC and their potential as clinical diagnostic markers, studies on circRNAs and lncRNAs are still scarce. An increasing number of studies have confirmed that lncRNAs and circRNAs can be involved in disease onset and progression through multiple mechanisms; however, whether they have the same effect in ACC needs to be further explored. Finally, the application and research on the diagnosis and treatment of ncRNAs in ACC also require further attention. While the highly potential role of ncRNAs in tumorigenesis, malignant metastasis, and tumor drug resistance has been demonstrated, studies on the role of ncRNAs in tumors and their clinical relevance are still in the developmental stage.

## 5. Conclusion

Overall, this review summarized the expression patterns of miRNAs, lncRNAs and circRNAs and their molecular mechanisms, as well as the clinical applications for ACC. With the advancement in genome sequencing and the detection technologies, the pathophysiological mechanisms of ncRNAs in ACC will be elucidated in-depth, which may provide the theoretical basis of early diagnosis and therapy for ACC.

## Acknowledgements

All graphic figures were made with biorender.com. We would like to thank Editage for English language editing.

## Author contributions

**Conceptualization:** Changfen Xu, Peiyao Xu, Jiaqi Zhang, Aiwu Huang.

**Data curation:** Changfen Xu, Peiyao Xu, Jiaqi Zhang, Sheng He, Tingting Hua, Aiwu Huang.

**Formal analysis:** Changfen Xu, Peiyao Xu, Sheng He, Tingting Hua, Aiwu Huang.

**Investigation:** Changfen Xu, Peiyao Xu, Aiwu Huang.

**Methodology:** Changfen Xu, Aiwu Huang.

**Resources:** Sheng He.

**Software:** Peiyao Xu, Sheng He.

**Visualization:** Peiyao Xu, Jiaqi Zhang, Tingting Hua.

**Writing – original draft:** Changfen Xu, Tingting Hua, Aiwu Huang.
